# Fusing Specialized Surveys of Rare Populations to Larger Surveys for Generalized Inference: Cross-Sectional Survey Study

**DOI:** 10.2196/86059

**Published:** 2026-04-27

**Authors:** Karilynn M Rockhill, Elizabeth A Bemis, Nicole Schow, Heather A Olsen, Kyle Beekman, Evelyn J Fox, Andrew A Monte, Joshua C Black

**Affiliations:** 1 Rocky Mountain Poison & Drug Safety, Denver Health and Hospital Authority, 777 Bannock Street, M/C 0180, Denver, CO, 80204, United States, 1 3033891652; 2 Emergency Medicine Department, University of Colorado School of Medicine, Aurora, CO, United States

**Keywords:** online survey, data fusion, psychedelics, statistical adjustment, psilocybin, lysergic acid diethylamide, LSD, methylenedioxymethamphetamine, MDMA, representativeness

## Abstract

**Background:**

Mainstays of pharmacoepidemiology are large, representative, behavioral surveys, which focus on many drugs with few detailed behaviors. Smaller, targeted studies measure drug-specific patterns but without explicit generalizability assumptions; the evidence generated is narrow.

**Objective:**

In this cross-sectional survey study, we outline an estimation framework based on data fusion and combine two surveys: a representative, anchor survey and an enriched survey about psychedelic drugs in the United States. Application of calibration weighting transports estimates from the enriched survey to the anchor survey.

**Methods:**

The psychedelic-focused enriched survey was sampled twice from a commercial online panel of adults from April 19 to June 25, 2024, (n=2306; 40.4% female, 33.1 y median age) and January 24 to March 21, 2025 (n=2023; 39.6% female, 35.2 y median age). The anchor survey was sampled twice from a different online panel from March 13 to May 6 2024 (n=28,679 total; 2430 using psychedelics) and 14 August to 9 October 2024 (n=29,040 total; 2309 using psychedelics). Internal consistency (transport bias, the absolute difference between the weighted estimates from the anchor survey and weighted fused survey) and external validity (root-mean-square error, RMSE, of self-reported demographic, health, and substance use estimates to probability-based benchmarks) metrics were calculated. The methodology was applied to estimate reasons for using specific psychedelic drugs.

**Results:**

Without adjustments, the enriched surveys had lower percentages of male and White respondents, lower self-perceived health, and higher cigarette use. A total of 2048 weighting schemes were tested, with good internal consistency. Average transport biases with the final weighting scheme were: demographics, 0.09 percentage points; health characteristics, 0.35 percentage points; and substance use, 0.22 percentage points. Estimates after fusion were externally consistent with benchmarks. RMSE increased by 3.3% for demographic estimates (1.82 unweighted to 1.88 weighted); larger decreases were observed for health RMSE (7.30 to 3.38, 53.7% decrease) and for substance use RMSE (6.56 to 6.03, 8.1% decrease). Alcohol use substantially increased the RMSE, likely due to question differences (without alcohol, the RMSE decreased from 6.03 to 1.55). Using the fused dataset, recreational use of psilocybin (92.9%, 95% CI 91.1, 94.7), LSD (93.2%, CI 90.1, 96.4, and MDMA (93.3%, 91.0, 95.6) was more common than medical use (30.9%, CI 27.6, 34.2; 26.4%, CI 21.1, 31.7; and 21.1%, CI 17.5, 24.7, respectively).

**Conclusions:**

Building upon past data fusion research, this study fused two surveys for the purpose of surveillance. This methodology, termed the “fused survey design,” is a rigorous but accessible approach for surveilling rare behaviors like drug use, and we demonstrated constructs absent from anchor surveys may be measured with generalizable inference. This expands the surveillance epidemiology toolbox, giving researchers an actionable process to field enriched surveys with specialized questions that would be impractical to add to larger surveys due to space constraints and respondent fatigue.

## Introduction

### Problem

Mainstays of epidemiology and surveillance of public health trends are large, representative surveys of human behavior. In pharmacoepidemiology, these surveys focus on drug use behaviors and health outcomes, of which there are several surveys across many countries [1-6]. These surveys study many drugs and thereby include only a few questions per drug; in essence, prioritizing breadth of knowledge over depth of knowledge about specific drugs. Large surveys provide essential information about substance use prevalence and correlates with demographics and predictors of health across multiple drug classes [7-11]. Smaller, targeted studies frequently measure drug-specific patterns, generating insight into how specific drugs are used in context, perceptions of use and harm reduction, and subgroup analyses that are challenging to field at scale [12-16]. Results from these smaller surveys are meaningful within the context of their sampling frame, but without explicit assumptions detailing the generalizability, the evidence generated may be narrow.

### Review of Relevant Scholarship

The lack of generalizable estimates from smaller surveys presents a specific problem for surveilling psychedelic drug use. While use patterns are changing globally across many classes [17], psychedelic drugs, particularly psilocybin have rapidly grown in popularity [18,19]. From 2015 to 2022, the Global Drug Survey demonstrated increased prevalence of use of psilocybin, lysergic acid diethylamide (LSD), 3,4-methylenedioxymethamphetamine (MDMA), ketamine (a dissociative drug with psychedelic properties), and to a lesser extent 4-bromo-2,5-dimethoxyphenethylamine across Europe, the United States, Australia, and Brazil [1]. Policy makers will likely need to adapt regulations, such as has already occurred across the United States [20], presenting an immediate surveillance need to study the motivations, experiences, and attendant health benefits and risks of targeted subgroups in a generalizable framework [21,22]. However, despite increasing use and popularity, prevalence remains rare compared to other substances like alcohol, tobacco, or cannabis, and there is a vast spectrum of behaviors that may influence health and safety. In response, psychedelic drugs have become a subject for targeted survey studies using anonymous online surveys [21,23,24], which may be advantageous for encouraging accurate reporting of stigmatized behaviors. However, online recruitment suffers this problem of lack of generalizability to target populations of interest [25]. Therefore, solving the generalizability problem would substantially improve surveillance capacity for psychedelic drugs, presenting better data for policy-making [22].

Data fusion and transport weighting methodology can overcome this problem [26-28]. Selection bias between data sources must be corrected to successfully fuse data if descriptive inference is to be generalizable, and, if causal inference is the objective, selection diagrams can decode the necessary assumptions [26]. Prior research demonstrates the utility of fusing multiple surveys to enhance data collection. An early example fused two surveys to improve accuracy of prevalence estimates of diabetes and cardiovascular disease [29]. Analogous data fusion procedures have been suggested for nonprobability surveys to fill gaps in low response-rate probability-based surveys [30], used to compare different generations of veterans’ health and social wellbeing [31], and used to combine multiple survey data for depression diagnoses [32]. In these cases, the topic under study was prevalent in the applicable populations. In the present study, we expand upon this foundation, demonstrating valid inference for combining data from different surveys to study rare subpopulations.

### Study Objectives

Our objectives were to: (1) describe the application of data fusion to population survey inference, (2) apply a quantitative framework for testing external validity of estimates produced from fusing surveys, and (3) demonstrate a practical application by estimating reasons for psychedelic drug use in the United States by fusing a small, targeted survey to a large, representative survey.

## Methods

### Design Overview and Assumptions

**Figure 1.**
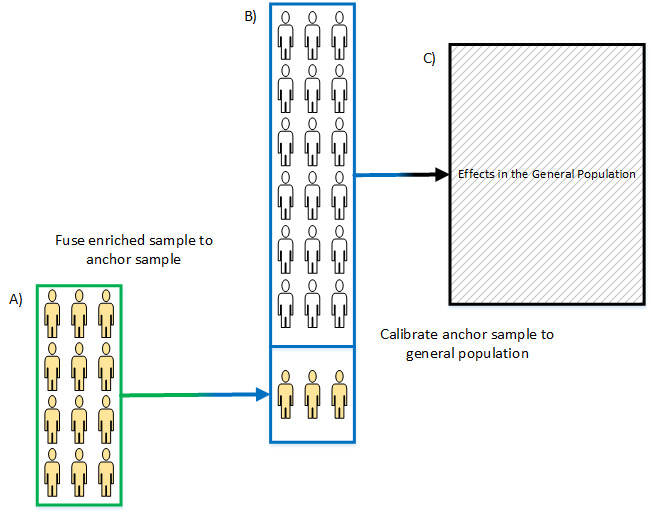
Conceptual diagram of fusing an enriched sample with an anchor sample to create generalizable estimates. A is an enriched sample collected with selection bias due to the targeted recruitment regarding the specialized content. B is an anchor sample collected with selection bias but without specific intent upon recruiting a profile of behavior. C is the target population where inference is intended.

We created a two-step data fusion inspired approach to combine surveys for enhanced inference [26,27,33]. Figure 1 outlines the conceptual framework describing two survey samples, collected separately, with different recruitment strategies, with the intent of ultimately generating inference to the general population (Figure 1C, black outline). A large general survey without enriched recruitment (Figure 1B, blue outline) is collected with a well-established sampling frame; we term this the “anchor survey.” The smaller, targeted survey is collected, with additional selection biases due to targeted sampling; we term this the “enriched survey” (Figure 1A, green outline).

For clarity, we define terms describing related phenomena which have slightly different meanings. In this context, “data fusion” refers to the process of combining two surveys into a single dataset for analysis. “Calibration” refers to the statistical process of adjusting weights so that variable distributions match between the surveys or benchmark population. “Transport weights” refer to individual-level values that, when used to generate statistical estimates, calibrate the enriched sample to the anchor sample; “transport weighting scheme” refers to the set of variables used to create the transport weights.

This framework partitions correction of selection bias into two steps requiring three primary assumptions. First, intersurvey calibration makes the enriched sample match the population corrected values of the analogous subsample in the anchor survey (shown in yellow in Figure 1B). Accurate calibration (A to B in Figure 1) requires an assumption that all factors that induce a selection bias between surveys are accounted for and measurable between the two surveys. If a factor produces differential probability of recruitment between the two surveys and is associated with the outcome, then this would induce a bias to estimates. In our practical application, we verified this assumption by examining internal consistency between the fused survey and anchor survey across a range of metrics likely associated with psychedelic use. Second, the fused survey is calibrated for population inference, which assumes generalizing weights are identifiable (B to C in Figure 1). We verify this assumption in our practical application by examining external validity across a range of metrics found in benchmark surveys. Both steps use generalized raking to calibrate [34]. Generalized raking is well suited for this because many variables associated with selection bias need adjustment and raking does not require complete cross-stratified distributions. This approach also assumes differential measurement bias between samples is negligible, and that any difference that could be observed was due to differences in selection.

### Data Sources and Sampling Procedures

The enriched survey, the National Survey Investigating Hallucinogenic Trends (NSIHT), collected detailed information about last 12-month use of psychedelic drugs. This enriched survey was fused to an existing, well-established anchor survey of the general adult population, the Survey of Non-Medical Use of Prescription Drugs (NMURx) [3]. The study plan with design details was pre-registered at the Open Science Framework, but is briefly summarized here and deviations are described in the supplement [35].

The psychedelic-focused enriched survey was sampled from a commercial online panel administered by the commercial research company YouGov (Palo Alto, California, US) in two waves, from April 19 to June 25 , 2024 and from January 24 to March 21, 2025. Respondents were required to answer all questions; therefore, there was no missing data. The first wave was used to develop and select the least biased transport weights, and the second wave replicated the results and tested external validity. For both waves, participants consented to being surveyed, were 18‐110 years old, and self-reported using at least one psychedelic drug in the last 12 months. Quotas based on age by biological sex and region of residence balanced initial distribution of these groups. The recruitment pool was a group of individuals who take surveys for modest compensation on a variety of topics (eg, health, sports, politics, consumer products) and are a neutral pool of participants with regards to drug use. As an online survey, respondents demonstrating careless responding (ie, failing to give necessary attention to answering questions) [36] were removed to reduce measurement error from misclassification.

The anchor survey, NMURx, was recruited from Kantar’s Profiles division (London, United Kingdom) proprietary online panel network, which has an established set of survey weights previously validated for drug use estimates [3]. As with the enriched survey, respondents were 18‐110 years old, and were required to answer all questions. This survey included an identifiable subsample who used psychedelic drugs in the last 12 months. Much like other large drug use surveys, NMURx prioritized breadth, not depth of psychedelic use. The survey was fielded twice, from March 13 to May 6 and from August 14 to October 9, 2024. The first wave was used to develop and select least biased transport weights and the second used to replicate the results and generate final estimates.

### Two-Step Calibration

In the fusion step (1A to 1B in Figure 1), intersurvey recruitment differences, and therefore differential selection forces, were calibrated using generalized raking. We took an empirical approach to satisfying the assumption that selection differences were accounted for in the transport weighting scheme, because we did not *a priori* know which variables may best represent selection differences between surveys. Using a previously published framework for studying psychedelic use in the real world as a guide [22], several possible variables were included on the enriched survey that may represent selection differences specifically related to psychedelic use. We included variables describing demographics, recent substance use, health, spirituality, and trust. Through multiple variable sets, we iterated testing how well each reduced overall bias relative to federal benchmark estimates. Variables in the enriched survey were calibrated to match the weighted values of the psychedelic use subsample in the anchor survey, resulting in transport weights that correct for composition differences between the two surveys (shown in yellow in Figure 1B). To test different weighting schemes, variables were split into two groups: required and iterated (Table 1). Required variables included a base set of demographic and health variables that we have demonstrated are important to correct for when using online panel data [3]. Iterated variables are those we hypothesized may differ between surveys and drive selection differences. Each combination of the iterated variables with the full set of required variables was tested (total of 2048 schemes) with required 0.1 percentage point convergence. The enriched survey with transport weights then replaced the entire anchor sub-sample, where the weighted marginal distributions of variables matched between enriched and anchor surveys. This is the fused survey.

**Table 1.** Known and hypothesized variables to correct for selection bias.

Required variables (Known)	Iterated variables (Hypothesized)
Age^a^	Survey language
Gender^a^	Income
Census division^a^	Marital status
Limited in daily activities^a^	Self-assessment of general health^a^
Current cigarette use^a^	Trust in people^a^
Ethnicity^a^	Spirituality
Self-reported race^a^	Number of psychedelic drugs used in last year
	Number of illicit drugs used in last year
	Number of psychoactive prescription drug classes used on a regular basis last year
	Number of lifetime diagnoses of mental health illnesses
	Drug Abuse Screening Test (DAST-10) severity score

a Included in final transport weighting scheme to create the fused sample

In the generalizing step (1B to 1C in Figure 1), differences in selection between the fused survey and the national population were calibrated. Here, we used our previously validated method of calibrating to national benchmarks [3]. Briefly, three demographic and two health benchmarks were raked against estimates from federal benchmark surveys—the American Community Survey and National Health Interview Survey. After the two calibration steps, final estimates from the fused survey were generalizable to the target population, adults in the US.

### Statistical Analysis

Validity of the fusion was evaluated in two ways. Internal consistency was assessed by comparing an independent set of evaluation variables, i (Table S1 in Multimedia Appendix 1):


(1) $$Y_{i}=|({\hat{X}}_{i}^{A}-{\hat{X}}_{i}^{F})$$

where, transport bias$Y_{i}$ represents the absolute difference between the weighted population estimate from the anchor survey (${{\hat{X}}_{}}_{i}^{A})$ to the weighted population estimate from the fused survey (${\hat{X}}_{i}^{F}$). $Y_{i}$ would equal zero if no transport bias was introduced by the fusion. A parsimonious transport weighting scheme was chosen that minimized the mean $Y_{i}$ across evaluation variables across three categories (demographic, health, and substance use). Internal consistency was measured for full population estimates and for the sub-population of adults reporting last 12-month psychedelic drug use. The selected weighting scheme was then applied to the independent second wave to test replicability. Weight stability was assessed by comparing convergence behavior, weight distributions, and the effective sample size (ESS) ratio. The ESS ratio is interpreted as how much of the sample was effectively retained after fusion, relative to a hypothetical simple random sample with equal variance.

To assess external validity, the population estimates from the independent second wave were compared to benchmark estimates from US federal surveys (Table S2 in Multimedia Appendix 1). One national survey was selected as the “gold standard,” but multiple estimates are shown to demonstrate variability across probability surveys where available. The root-mean-square error (RMSE) of the benchmark estimates, k, was calculated as:


(2) $$RMSE(\text{weighted})=\sqrt{\sum_{k=1}^{K}\frac{({\hat{X}}_{k}^{GS}-{\hat{X}}_{k}^{F}{)}^{2}}{K}}$$

(3) $$RMSE(\text{unweighted})=\sqrt{\sum_{k=1}^{K}\frac{({\hat{X}}_{k}^{GS}-{\hat{X}}_{k}^{E}{)}^{2}}{K}}$$

where the estimates from the gold standard (${{\hat{X}}_{}}_{k}^{GS})$ and the estimates from the weighted fused sample (${\hat{X}}_{k}^{F}$) and unweighted, not transported, enriched survey (${\hat{X}}_{k}^{E}$) were stratified by category.

Finally, the percentage and 95% CIs for reason of use among adults who used three psychedelic substances (ie, psilocybin, LSD, and MDMA) in the last 12 months were estimated by Taylor series expansion to demonstrate new information produced through the fusion method. All analyses were conducted using SAS (version 9.4; SAS Institute Inc.). This study is reported following the Journal Article Reporting Standards for Quantitative Research (JARS-Quant) guidelines [37].

### Ethical Considerations

The Colorado Multiple Institutional Review Board first approved the anchor sample study on July 5, 2016 (#16‐0922) and the enriched sample study on February 02, 2024 (#23‐2426), allowing ongoing secondary analyses of these data without additional consent. All participants consented to being surveyed in the online questionnaires, the survey was confidentially administered, and study datasets were deidentified by the panel companies prior to transferring them to the researchers. No identification of individual participants in any parts of the manuscript or supplementary material is possible. In both panels, participants were compensated with points, which could be redeemed for gift cards of approximately US $5.

## Results

### Selecting a Transport Weighting Scheme

The first wave of the enriched survey invited 18,907 panelists (Figure S1 in Multimedia Appendix 1), of which 16,423 participated (16,423/18,907; 86.9% participation rate) and 15,430 completed it (15,430/16,423; 94.0% completion rate). The final enriched sample included 2306 respondents. The enriched survey was 40.4% female (932/2306) and had a median age of 33.1 (interquartile range [IQR]: 25.8‐39.7). The first wave of the anchor survey had a total sample of 28,679 respondents, where 2430 respondents reported using at least one psychedelic drug in the last 12 months and were weighted to represent 12.7 million adults (95% CI 11.9, 13.4). Table S3 in Multimedia Appendix 1 compares the enriched survey to the anchor survey weighted subpopulation using psychedelic drugs. Without adjustments, the enriched survey had lower percentages of male and White respondents, lower self-perceived health, and higher current cigarette use.

**Figure 2.**
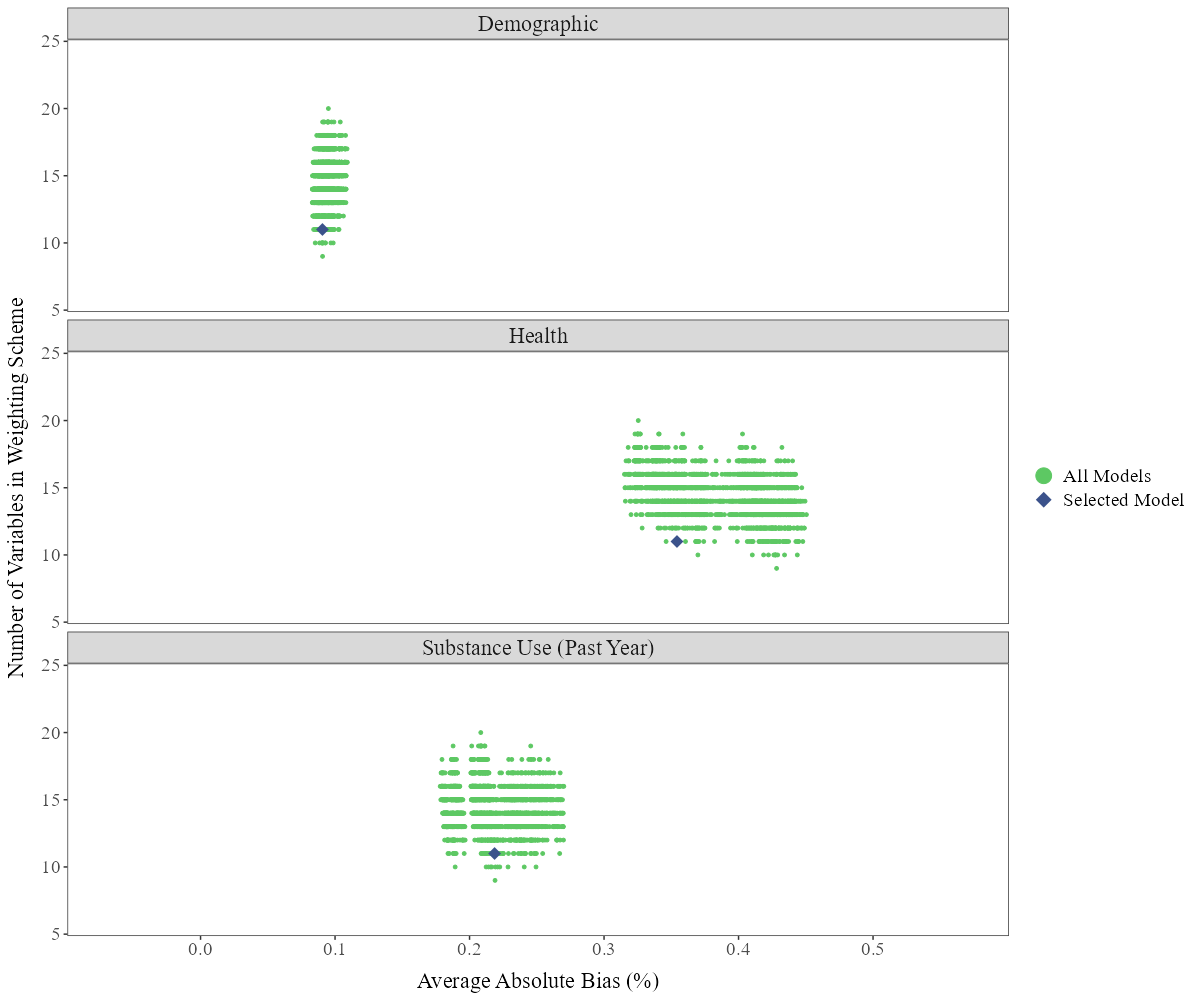
Average transport bias in evaluation estimates, internal consistency in fused dataset. Internal consistency was tested using data from the first half of 2024. Green dots represent average transport bias across all iterations of the transport weighting schemes. Purple diamond is the selected model, which was a parsimonious scheme with low bias across all three categories*.*

Fusion introduced minimal transport bias relative to the anchor survey, demonstrating good internal consistency (Figure 2). Across all weighting schemes, the absolute transport bias in demographic, health, and substance use metrics was less than 0.5 percentage points after fusion. The selected transport weighting scheme, shown by a diamond, was relatively parsimonious, including two variables (trust in people and self-assessment of general health) in addition to the required demographics. The average transport biases with the selected weighting scheme were: demographics, 0.09 percentage points; health characteristics, 0.35 percentage points; and substance use, 0.22 percentage points.

### Replicability and Weight Stability

The selected transport weighting scheme was then applied to the second wave of enriched survey where 36,592 panelists were invited (Figure S2 in Multimedia Appendix 1), of which 27,868 participated (27,868/36,592; 76.2% participation rate) and 25,842 completed it (25,842/27,868; 92.7% completion rate). The final enriched survey included 2023 respondents. This survey was 39.6% female (801/2,023) and had a median age of 35.2 (IQR 26.8‐44.4). The second wave of the anchor survey had a total sample of 29,040 respondents, where 2309 respondents reported using at least one psychedelic drug in the last 12-months and were weighted to represent 12.7 million adults (95% CI: 11.9, 13.5).

Fusion did not substantially perturb the demographics of the subpopulation using psychedelic drugs in either the first or second samples (Figures S3,and S4 in Multimedia Appendix 1). Geography, age, biologic sex, employment, and most health and substance use metrics were not perturbed by fusion. Health and substance use metrics had larger shifts in proportions after fusion compared to demographics, but shifts were not persistent when retested. Two metrics were consistently different across sample pairs. The fused sample had higher proportion of adults with high school or equivalent education and lower mental health treatment.

The weighting scheme converged in a similar number of iterations across waves (44 in Wave 1 vs 48 in Wave 2), indicating comparable optimization difficulty. Extreme weights were rare in both waves and did not increase in the second launch (three observations exceeded median+5×IQR in Wave 1 vs. two observations in Wave 2), suggesting little, if any, instability in the upper tail of the weight distribution. Transport weight distributions between waves had similar central tendencies and overall ranges (median 4888 vs 5,304; maximum 37,671 vs 34,793). Although the 99th percentile increased modestly in Wave 2 (14,384 vs 18,794), this change was not accompanied by notable increases in the maximum weight or the number of extreme weights and did not result in meaningful differences in ESS. ESS ratios were nearly identical across waves (0.78 in Wave 1 vs 0.75 in Wave 2), indicating stable transport weighting performance and no meaningful change in variance inflation in the second wave (Table 2).

**Table 2.** Weight stability assessment.

Weight characteristics	Wave 1, Early 2024	Wave 2, Late 2024
Mean (SD)	5498.31 (2914.28)	6275.43 (3616.25)
Minimum	422.06	451.88
Maximum	37,671.13	34,793.16
Median	4887.76	5304.47
Upper tail: 99%	14,383.51	18,794.18
Effective sample size ratio^a^	0.78	0.75

a Interpreted as how much of sample size effectively retained after weighting

### External Validity

**Figure 3.**
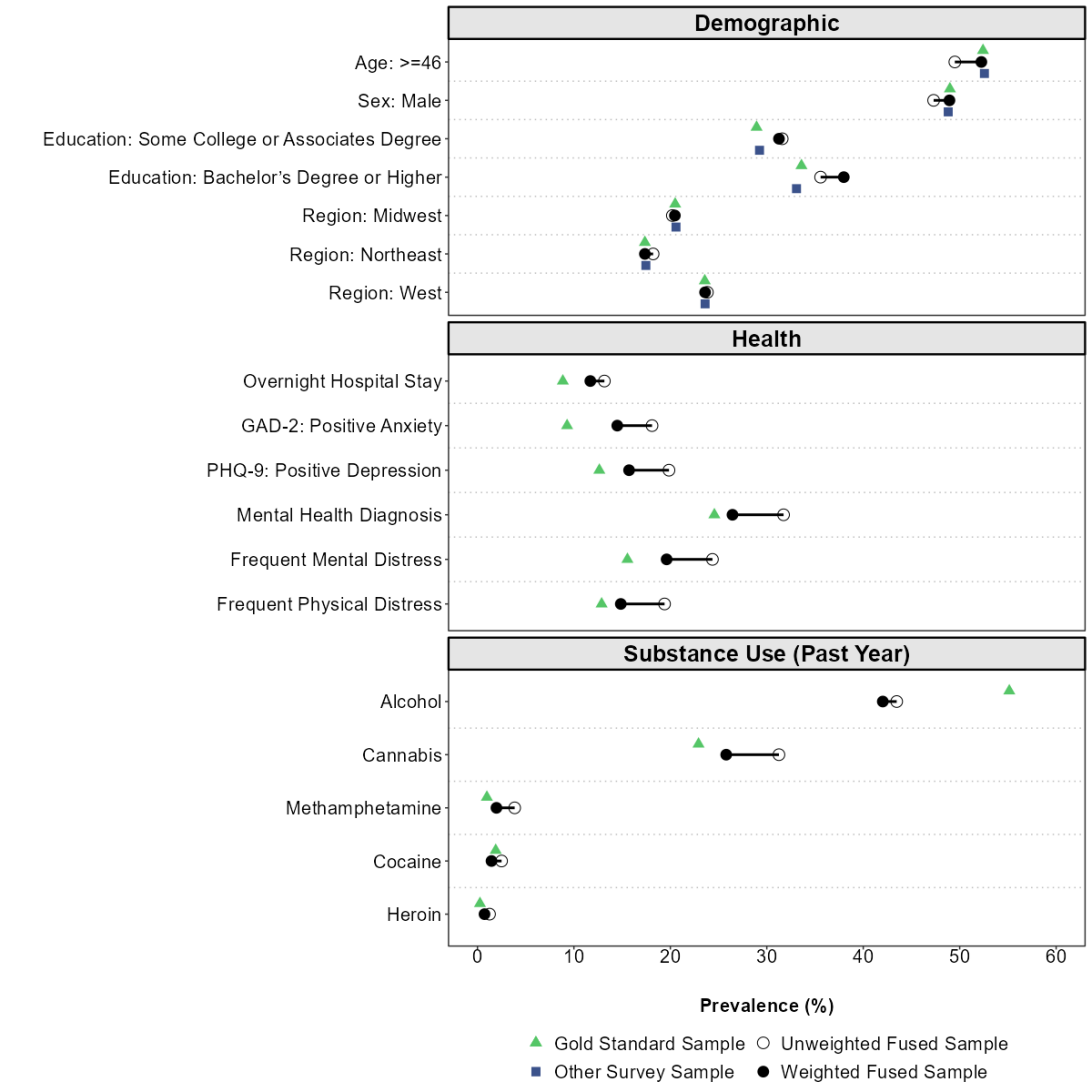
Impact of transport weighting on pre-defined benchmarks of demographics, health, and substance use estimates, external validity in fused survey. External validity was tested using data from the second half of 2024 and early 2025. US national gold standard benchmarks (triangles) and other survey (squares) estimates were substantially similar after transport weights (closed circle), and transport weighted values were corrected towards benchmark estimates in most health and substance use factors compared to unweighted estimates (open circle). Alcohol use questions were asked differently between the gold standard and this study. GAD-2, General Anxiety Disorder, 2 Item; PHQ-9, Patient Health Questionnaire, 9 Item.

For external validity, fusion did not substantially change the demographic RMSE (1.82 unweighted to 1.88 weighted, a 3.3% increase); larger decreases were observed for health RMSE (7.30 to 3.38, 53.7% decrease) and for substance use RMSE (6.56 to 6.03, 8.1% decrease). Final population estimates were similar to benchmark estimates (Table 3, Figure 3). Geography, age, sex, and spirituality [38] estimates in the fused survey were nearly identical to benchmarks. Small differences (<5% generally) persisted in race estimates, marital status, education, and employment status after weighting, though race questions were asked differently between surveys. The data fusion process corrected health estimates by increasing self-perceived health estimates and decreasing distress, hospital stays, and mental health estimates. After fusion, most substance use estimates were nearly equal to benchmark values. Notably, alcohol use estimates were substantially lower than the benchmark and substantially increased the RMSE; this is possibly due to differences in how the questions were asked between surveys (without alcohol, the RMSE decreased from 6.03 to 1.55).

**Table 3.** Comparison of estimates in fused dataset, after transport weighting, to external survey benchmarks, second half of 2024 and early 2025.

Characteristic	Wave 2, Late 2024 Unweighted fused % (95% CI)	Wave 2, Late 2024 Weighted fused % (95% CI)	Additional benchmarks % (95% CI)	Survey
Number of adults				
Sample size, n	28,754	28,754		
Estimated number of adults, in millions	—	266 (264, 268)		
Age (years)				
≥46	49.5 (48.9, 50.1)	52.2 (51.5, 53.0)	52.4 (52.3, 52.5)	ACS^a^
Median (IQR)	44.8 (34.4, 58.2)	46.5 (32.4, 62.5)	46.6 (31.8, 62.5)	ACS
Sex				
Female	52.7 (52.1, 53.3)	51.1 (50.3, 51.8)	51.0 (50.9, 51.1)	ACS
Colorado or Oregon resident				
Yes	3.3 (3.1, 3.5)	3.1 (2.9, 3.4)	3.1 (3.1, 3.1)	ACS
Ethnicity and race				
Hispanic or Latino	16.7 (16.2, 17.1)	17.2 (16.6, 17.7)	17.6 (17.5, 17.6)	ACS
Black or African American alone	12.5 (12.1, 12.8)	12.4 (11.9, 12.9)	11.8 (11.8, 11.9)	ACS
White alone	73.4 (72.8, 73.9)	72.4 (71.7, 73.1)	62.9 (62.8, 63.0)	ACS
Other race alone	11.2 (10.8, 11.5)	12.3 (11.8, 12.8)	14.3 (14.2, 14.3)	ACS
Multiple races	3.0 (2.8, 3.2)	2.9 (2.6, 3.1)	11.0 (11.0, 11.1)	ACS
Marital status				
Never married	29.0 (28.5, 29.5)	30.7 (30.0, 31.4)	31.1 (31.0, 31.1)	ACS
Now married	47.7 (47.1, 48.3)	47.6 (46.9, 48.3)	50.5 (50.4, 50.6)	ACS
Widowed, divorced, separated	23.3 (22.8, 23.8)	21.7 (21.1, 22.2)	18.4 (18.3, 18.5)	ACS
Limited in the kind or amount of work				
Yes	28.7 (28.2, 29.2)	19.4 (18.9, 19.9)	19.5 (18.9, 20.1)	NHIS^b^
Current employment				
Yes	58.1 (57.6, 58.7)	55.5 (54.8, 56.3)	60.9 (60.1, 61.6)	NHIS
General health				
Good, Very Good, or Excellent	81.5 (81.0, 81.9)	84.8 (84.3, 85.3)	84.9 (84.3, 85.4)	NHIS
Poor or Fair	18.5 (18.1, 19.0)	15.2 (14.7, 15.7)	15.1 (14.6, 15.7)	NHIS
Trust in People: 0-You can’t be too careful to 10-Most people can be trusted				
≥5 People Can Be Trusted	55.8 (55.3, 56.4)	56.3 (55.6, 57.0)		–^c^
Spirituality				
Not Spiritual or Don’t Know	20.3 (19.8, 20.8)	21.3 (20.7, 21.9)	21.0	Pew American Trends Panel 2023
Current cigarette use				
Yes	37.2 (36.7, 37.8)	10.8 (10.6, 11.1)	10.8 (10.3, 11.3)	NHIS
Number of psychedelic substances used past year				
1	4.1 (3.9, 4.3)	2.7 (2.5, 2.9)		–^c^
2	1.3 (1.2, 1.5)	0.9 (0.8, 1.0)		–^c^
3‐4	0.9 (0.8, 1.0)	0.6 (0.5, 0.7)		–^c^
≥5	0.2 (0.2, 0.3)	0.2 (0.1, 0.2)		–^c^
Number of illicit substances used past year				
1‐3	7.6 (7.3, 7.9)	4.3 (4.1, 4.6)		–^c^
≥4	1.5 (1.3, 1.6)	0.9 (0.8, 1.0)		–^c^
Number of psychoactive prescription drugs used on a regular basis past year				
≥3	4.2 (3.9, 4.4)	2.8 (2.6, 3.0)		–^c^
Number of lifetime mental health diagnoses				
≥3	10.7 (10.4, 11.1)	7.7 (7.4, 8.1)		–^c^
Problematic substance use				
≥3 DAST-10^d^ Severity Score	10.2 (9.9, 10.6)	6.5 (6.2, 6.8)		–^c^

a ACS: American Community Survey.

b NHIS: National Health Interview Survey.

c No available benchmark

d DAST: Drug Abuse Screening Test, 10 Item

Estimates presented in this table are not inclusive of all gold standard and other survey estimates calculated.

**Figure 4.**
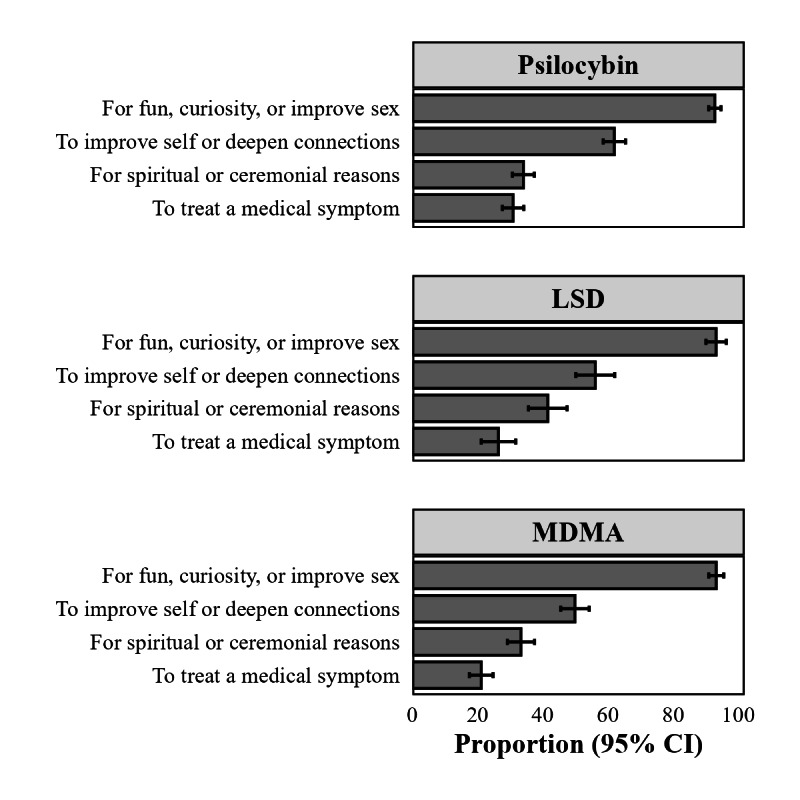
Reason for psychedelic substance use estimates using fused dataset, second half of 2024 and early 2025. Among adults in the United States who used each of the psychedelic substances below, the proportion who used for each reason differed with recreational use being most common while treating a medical symptom being least common. LSD,: lysergic acid diethylamide; MDMA: 3,4-methylenedioxymethamphetamine*.*

Fusion reduced the proportions of people using prescription, psychedelic, and illicit drugs and reduced the proportion with multiple mental health diagnoses. Using the transport weighting scheme, reasons for use differed by psychedelic drug. Recreational use of psilocybin (92.9%, CI: 91.1, 94.7), LSD (93.2%, CI 90.1, 96.4], and MDMA (93.3%, CI 91.0, 95.6) was more common than medical use (30.9%, CI 7.6, 34.2; 26.4%, CI 21.1, 31.7; and 21.1%, CI 17.5, 24.7, respectively). (Figure 4).

## Discussion

### Principal Findings

This study outlined a novel “fused survey design,” applied a quantitative framework to test external validity, and demonstrated a practical application by estimating reasons for using specific psychedelic drugs among the general US adult population. This method of fusing surveys generated internally consistent estimates of demographics, drug use, and health, which were replicated across two independently fielded survey waves. The methodology also reduced differences in prevalence and demographic estimates relative to national benchmarks, thereby demonstrating the method can create estimates externally valid to a well-defined target population. Application of the validated methodology showed recreational reasons for use were more common than medical reasons across three psychedelic substances (ie, psilocybin, LSD, MDMA).

### Specific Implications for Psychedelic Drug Research and Policy

In the United States, states have decriminalized and legalized medical access models, which has led to more people using [19]. Surveilling why people use psychedelic drugs would give policymakers evidence to enact regulations that supports safe, equitable, and effective access. These results suggest regulations that enable access should also account for substantial recreational use alongside medical use. The data fusion framework presented here produced generalizable estimates about a specific kind of behavior involving psychedelic drugs, which otherwise would not have been available in either large population surveys alone or in small, non-generalizable enriched surveys. The growing prevalence of psychedelic use presents an emergent health phenomenon where detailed depth of measurement is required to understand the health implications of increased use [22], and the fused survey design can address future questions in this research area.

### General Implications for Applying Data Fusion to Survey Samples

The framework presented in this work solves problems that arise when combining drug use using surveys for surveillance [30], and complements other survey-based applications of data fusion and transport weighting [39], which has historically focused on using surveys as targets for fusion with trial data, such as demonstrating the impact of a drug use interventions in more diverse populations [40,41]. The transport weighting scheme corrected for differences in demographic, health, and attitudinal perspectives of the enriched survey. These differences are correlated to medical outcomes of interest (eg, psychological symptoms, healthcare interactions), and if left uncorrected, would have introduced bias in the final estimates [42]. Nonprobability online panels are inherently not representative [25,30], and calibration of health and substance use was required to generate valid domain-specific estimates [25]. Data fusion would permit analysis between new populations not found in the enriched survey; for example in this study, characteristics between those who use psychedelics and those who use other medications without psychedelic use may be explored within the fused survey, but not within either survey alone. For psychedelic research, comparing to those who do not use is essential for developing effective policy and health recommendations, and drug-specific surveys which do not recruit those who do not use psychedelic drugs are unable to analyze that contrast [21].

This framework may be applied to a wide variety of research questions and may be particularly useful for rare subpopulations where direct sampling enrichment in a large survey is not feasible. Researchers should ensure that fusion samples are matched to similar subgroups of interest, that transport variables are measured as similarly as possible between enriched and anchor surveys, and that metrics are available to confirm the validity of the transport. Assumptions may not be directly testable, so demonstrating internal consistency and external validity, as has been done here, would strengthen results using this method. The methodology can also be used to fuse nonprobability surveys with probability-based surveys. Given the low response rate in many government surveys, in the US and elsewhere, nonprobability sampling may fill important gaps in surveillance in many disciplines, not just pharmacoepidemiology [30].

### Limitations

The assumption that there was no measurement bias was not directly tested. Our research group controlled both the anchor and enriched surveys, so all transport variables had the same question wording, similar digital interfaces, and skip logic. This strengthened the validity of the measurement assumption for this pilot. If measurements were not equivalent, then calibration approach may not fully account for selection bias, because measurement and selection bias would be conflated. This may result in over- or under-correction, depending on the nature of measurement mismatch. Questions used to test validity of fusion should also match. In our results, there were differences in how past year alcohol use was measured. In our surveys, it was a single question asking whether a person had 12 drinks in the past 12 months. In the NHIS benchmark, two questions asked about lifetime use then about past 12-month use [43]. The NHIS question also provided a list of types of drinks, which may have improved recall. Data fusion and validity testing are influenced by measurement differences [26], and researchers who use this method should minimize such differences.

While the methodology overcomes many inferential limitations, the survey remains a rare population survey, where subgroups within the rare populations (eg, Indigenous peoples using psychedelics) may still be infrequently sampled. Weighting cannot eliminate stochastic uncertainty from low sample size [44]. However, fusion methods may allow multiple survey sets to be combined, thereby increasing subgroup sample size. Recent advances weighting individual variables may enable additional transport options for subgroups [45]. Additionally, there were small residual differences between the fusion estimates and benchmarks, potentially due to interactions between variables not accounted for in calibration. While other unmeasured selection differences may be present, this study examined a wide variety of variables that may bias selection, and we selected a final scheme that minimized differences against a national benchmark in a data-driven and parsimonious manner. Future work may incorporate multi-level calibration, which may further reduce bias by accounting for such higher-order interactions [46]. Measurement differences in how questions were asked or fielding date differences may contribute. Finally, this method requires the subpopulations between samples be aligned. In this pilot work, the studies had slightly mismatched drug groups, where approximately 15% of the enriched sample endorsed a drug not examined in the anchor sample. These respondents were kept in the fused dataset for this pilot, which may result in unadjusted transport bias in estimates involving data about these drugs.

### Conclusion

Building upon past data fusion research, this study fused two surveys for the purpose of surveillance. This methodology, termed the “fused survey design,” is a rigorous but accessible approach for surveilling rare behaviors like drug use, and we demonstrated constructs absent from anchor surveys may be measured with generalizable inference. This expands the surveillance epidemiology toolbox, giving researchers an actionable process to field enriched surveys with specialized questions that would be impractical to add to larger surveys due to space constraints and respondent fatigue.

## Supplementary material

10.2196/86059 Multimedia Appendix 1 Additional results.

## Abbreviations

ACS: American Community Survey
DAST-10: Drug Abuse Screening Test, 10 Item
ESS: Effective sample size
GAD-2: General Anxiety Disorder, 2 Item
JARS-Quant: Journal Article Reporting Standards for Quantitative Research
LSD: lysergic acid diethylamide
MDMA: 3,4-methylenedioxymethamphetamine
NHIS: National Health Interview Survey
NMURx: Survey of Non-Medical Use of Prescription Drugs
NSIHT: National Survey Investigating Hallucinogenic Trends
PHQ-9: Patient Health Questionnaire, 9 Item
RMSE: root-mean-square error

## References

[1] Winstock A, Munksgaard R, Davies E, Ferris J, ZhuParris A, Barratt M (2022). Global Drug Survey.

[2] (2024). Behavioral Health Statistics and Quality, Substance Abuse and Mental Health Services Administration.

[3] Black JC, Rockhill K, Forber A (2019). An online survey for pharmacoepidemiological investigation (Survey of Non-Medical Use of Prescription Drugs Program): validation study. J Med Internet Res.

[4] Drug misuse in england and wales: year ending march 2024. ONS Centre for Crime and Justice.

[5] European web survey on drugs. Lisbon, Portugal: European Union Drugs Agency.

[6] Canadian Substance Use Survey (CSUS): summary of results for 2023.

[7] Chen JJ, Berg CJ, Yang YT (2026). Hallucinogen use in the United States, 2021-2023: diverging trends and subgroup patterns. Drug Alcohol Depend Rep.

[8] Cherro M, Itani H, Ghossoub E, Maalouf F (2025). Predictors of suicide attempts among adolescents with suicidal ideations and a plan: results from the National Survey on Drug use and Health (NSDUH). PLoS One.

[9] Han B, Jones CM, Volkow ND (2025). Prevalence of cannabis consumption methods among people with medically recommended and nonmedical cannabis use in the United States. Addiction.

[10] Han B, Jones CM, Volkow ND (2025). Prescription stimulant use, misuse, and use disorder among US adults aged 18 to 64 years. JAMA Psychiatry.

[11] Stroud LR, Gautam P, Jao NC, Sharma E (2025). Menthol cigarette use in US pregnancies: prevalence, racial and ethnic disparities, and associated characteristics from the 2010-2019 National Survey on Drug Use and Health (NSDUH). Nicotine Tob Res.

[12] Lenton S, Potter G, Fortin D (2025). Growing practices and the use of potentially harmful chemical additives from a web survey of mainly small-scale cannabis growers in 18 countries. Int J Drug Policy.

[13] Marotta PL, Biaid M, Heimer R (2025). Rural providers’ attitudes toward integrating harm reduction strategies and PrEP prescribing into rural primary care settings in the US. Southeast and Midwest. Addict Sci Clin Pract.

[14] Music J, Sterling B, Charlebois S, Goedhart C (2024). Comparison of perceptions in Canada and USA regarding cannabis and edibles. J Cannabis Res.

[15] Örüm MH, Kapıcı Y, Sönmez D (2025). Practices and attitudes of adult psychiatrists regarding methamphetamine-associated psychotic disorder: an internet based survey conducted in Turkey. BMC Health Serv Res.

[16] Wilkins C, Romeo J, Rychert M, Graydon-Guy T (2024). Exploring the substitution of cannabis for alcohol and other drugs among a large convenience sample of people who use cannabis. Harm Reduct J.

[17] Zhu DT, Kwon YIC, Lai A, Park AMG, Barnes AJ, Chapman DA (2025). Global burden of disease due to opioid, amphetamine, cocaine, and cannabis use disorders, 1990-2021: a systematic analysis for the Global Burden of Disease Study 2021. PLoS One.

[18] Bhave A (2025). Magic of the mushrooms: effects of psilocybin decriminalization. J Psychoactive Drugs.

[19] Black JC, Bau GE, Cook RR, Bemis EA, Monte AA (2026). Psilocybin trends in states that decriminalized use. JAMA.

[20] Siegel JS, Daily JE, Perry DA, Nicol GE (2023). Psychedelic drug legislative reform and legalization in the US. JAMA Psychiatry.

[21] Lake S, Lucas P (2024). The Global Psychedelic Survey: consumer characteristics, patterns of use, and access in primarily anglophone regions around the world. Int J Drug Policy.

[22] Black JC, Monte AA, Dasgupta N (2024). Optimizing real-world benefit and risk of new psychedelic medications: the need for innovative postmarket surveillance. Nat Mental Health.

[23] Kopra EI, Ferris JA, Winstock AR, Kuypers KP, Young AH, Rucker JJ (2023). Investigation of self-treatment with lysergic acid diethylamide and psilocybin mushrooms: findings from the Global Drug Survey 2020. J Psychopharmacol.

[24] (2024). Use of psychedelic substances in the United States. Results from the National Survey Investigating Hallucinogenic Trends (NSIHT).

[25] Mercer AW, Kreuter F, Keeter S, Stuart EA (2017). Theory and practice in nonprobability surveys. Public Opin Q.

[26] Bareinboim E, Pearl J (2016). Causal inference and the data-fusion problem. Proc Natl Acad Sci U S A.

[27] Josey KP, Yang F, Ghosh D, Raghavan S (2022). A calibration approach to transportability and data-fusion with observational data. Stat Med.

[28] Yang S, Ding P (2020). Combining multiple observational data sources to estimate causal effects. J Am Stat Assoc.

[29] Aluja-Banet T, Daunis-I-Estadella J, Brunsó N, Mompart-Penina A (2015). Improving prevalence estimation through data fusion: methods and validation. BMC Med Inform Decis Mak.

[30] Si Y, Wagner JR, Kessler RC (2026). Probability, probability-based, and nonprobability surveys in psychiatric epidemiological research. JAMA Psychiatry.

[31] Vogt D, Borowski S, Maguen S (2022). Strengths and vulnerabilities: comparing post-9/11 U.S. veterans’ and non-veterans’ perceptions of health and broader well-being. SSM Popul Health.

[32] Lee S, Lee S, Lee J, Jo YT, Park E, Cha J (2024). Fusion of multiple self-diagnostic questionnaires into optimal diagnostic cut-offs and factor analysis for depression characterization of the Korean university student group. BMC Psychiatry.

[33] Pearl J, Bareinboim E (2022). External Validity: From Do-Calculus to Transportability Across Populations Probabilistic and Causal Inference: The Works of Judea.

[34] Deville JC, Särndal CE, Sautory O (1993). Generalized Raking Procedures in Survey Sampling. J Am Stat Assoc.

[35] Rockhill K, Bemis EA, Schow N, Beekman K, Olsen HA, Fox EJ (2025). Establishment of External Validity of the National Survey Investigating Hallucinogenic Trends (NSIHT).

[36] Ward MK, Meade AW (2023). Dealing with Careless Responding in Survey Data: Prevention, Identification, and Recommended Best Practices. Annu Rev Psychol.

[37] Appelbaum M, Cooper H, Kline RB, Mayo-Wilson E, Nezu AM, Rao SM (2018). Journal article reporting standards for quantitative research in psychology: The APA Publications and Communications Board task force report. Am Psychol.

[38] (2023). Spirituality Among Americans.

[39] Vuong Q, Metcalfe RK, Ling A, Ackerman B, Inoue K, Park JJ (2025). Systematic review of applied transportability and generalizability analyses: A landscape analysis. Ann Epidemiol.

[40] Ackerman B, Lesko CR, Siddique J, Susukida R, Stuart EA (2021). Generalizing randomized trial findings to a target population using complex survey population data. Stat Med.

[41] Inoue K, Hsu W (2024). Transportability analysis-a tool for extending trial results to a representative target population. JAMA Netw Open.

[42] Black JC, Rockhill KM, Schow N, Monte AA (2025). Proactive bias mitigation when using online survey panels for self-reported use of illicitly manufactured fentanyl in the general adult population. JAMA Health Forum.

[43] (2023). 2023 NHIS Questionnaires, Datasets, and Documentation.

[44] Elvira V, Martino L, Robert CP (2022). Rethinking the effective sample size. Int Statistical Rev.

[45] Tripet A, Tillé Y (2025). Calibration and optimal transport approaches for harmonizing survey weights. Stat Methods Appl.

[46] Ben-Michael E, Feller A, Hartman E (2024). Multilevel calibration weighting for survey data. Polit Anal.

