# Supplementary material for: Fusing Specialized Surveys of Rare Populations to Larger Surveys for Generalized Inference: Cross-Sectional Survey Study
Source: J Med Internet Res. 2026 Apr 27;28:e86059. doi: 10.2196/86059 (PMC13120540; doi:10.2196/86059)
Supplement: Multimedia Appendix 1 [file jmir-v28-e86059-s001.docx]

**Supplemental Information**

**Supplemental Table 1. Independent set of evaluation variables used to assess internal consistency after data fusion**

| **Category** | **Variables Assessed for Internal Consistency** |
| --- | --- |
| Demographic | Age: binary |
|  | Sex |
|  | Education |
|  | Region of residence |
|  | Psychedelic decriminalization based on state of residence |
|  | Employment |
| Health | Frequent mental distress |
|  | Frequent physical distress |
|  | Any drug or alcohol treatment |
|  | Any outpatient mental health treatment |
| Substance Use (Last Year) | Alcohol |
|  | Cannabis |
|  | Methamphetamine |
|  | Cocaine |
|  | Heroin |

**Supplemental Table 2. Independent set of benchmark variables used to assess external consistency of data fusion process**

| **Type** | **External Benchmark Variable** | **Gold Standard Survey** | **Other Surveys** |
| --- | --- | --- | --- |
| Demographic | Age categories | ACS | NHIS |
|  | Sex |  |  |
|  | Education |  |  |
|  | Region of residence |  |  |
| Health | Frequent mental distress | BRFSS |  |
|  | Frequent physical distress |  |  |
|  | Any overnight hospital stay | NHIS |  |
|  | Any mental health lifetime diagnoses |  |  |
|  | General Anxiety Disorder (GAD-2) |  |  |
|  | Patient Health Questionnaire (PHQ-9) | NHANES |  |
| Substance Use (Past Year) | Alcohol | NHIS |  |
|  | Cannabis | NSDUH |  |
|  | Methamphetamine |  |  |
|  | Cocaine |  |  |
|  | Heroin |  |  |

*Abbreviations:* *ACS, American Community Survey; BRFSS, Behavioral Risk Factor Surveillance System; NHANES, National Health and Nutrition Examination Survey; NHIS, National Health Interview Survey; NSDUH, National Survey on Drug Use and Health*

One additional benchmark was included due to its relevance to psychedelic drug use, which is an estimate of spirituality from the Pew Research Center.

**Supplemental Figure 1. Recruitment flowchart for first wave of the enriched sample used in development of transport weights**


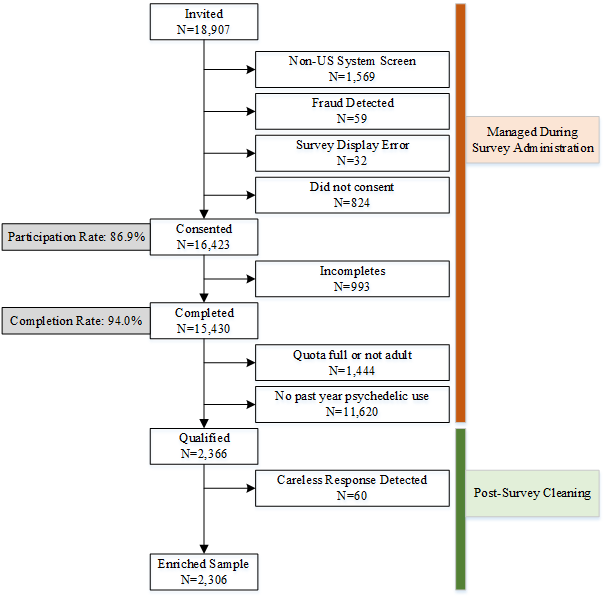


**Supplemental Table 3. Characteristics of the enriched sample to the anchor sample weighted subpopulation using psychedelic drugs, first wave.**

| **Characteristic** | **Enriched Sample**  **Unweighted**  **%** | **Anchor Psychedelic Sample**  **Weighted**  **% (95% CI)** |
| --- | --- | --- |
| **N** | | |
| Sample Size, n | 2,306 | 2,430 |
| Number of adults, weighted in millions (95% CI) | NA | 12.7 (11.9, 13.4) |
| **Age (years)** | | |
| Median, IQR | 33.1 (25.8, 39.7) | 33.3 (26.8, 40.8) |
| **Sex** | | |
| Female | 40.4 | 35.0 (32.2, 37.8) |
| **Gender Identity** | | |
| Female | 39.3 | 35.3 (32.5, 38.1) |
| Male | 57.1 | 63.0 (60.1, 65.8) |
| Any Other | 3.6 | 1.8 (1.0, 2.5) |
| **Ethnicity and Race** | | |
| Hispanic or Latino | 20.2 | 20.7 (18.2, 23.2) |
| Black or African American Alone | 19.2 | 11.6 (9.5, 13.7) |
| White Alone | 67.9 | 74.7 (71.9, 77.5) |
| Any Other Race Alone | 8.8 | 10.0 (8.0, 12.0) |
| Multiple Races | 4.2 | 3.7 (2.4, 5.0) |
| **Education** | | |
| Bachelor’s Degree or Higher or Trade School | 47.5 | 50.2 (47.2, 53.2) |
| High School or GED | 23.0 | 16.7 (14.4, 19.0) |
| Less than High School | 3.5 | 4.5 (3.3, 5.7) |
| Some College or Associate’s Degree | 26.0 | 28.6 (25.8, 31.4) |
| **Income (USD)** | | |
| ≥ $75,000 | 52.4 | 47.0 (44.0, 50.0) |
| **General Health** | | |
| Good, Very Good, or Excellent | 83.5 | 89.5 (87.7, 91.3) |
| Poor or Fair | 16.5 | 10.5 (8.7, 12.3) |
| **Limited in the Kind or Amount of Work** | | |
| Yes | 24.3 | 29.6 (27.1, 32.1) |
| **Current Cigarette Use** | | |
| Yes | 46.4 | 38.6 (36.1, 41.1) |
| **Problematic Substance Use** | | |
| ≥3 DAST-10 Severity Score | 42.8 | 42.0 (39.1, 45.0) |
| **Region** | | |
| Midwest | 13.9 | 16.2 (14.0, 18.4) |
| Northeast | 21.1 | 18.0 (15.7, 20.2) |
| South | 35.6 | 35.0 (32.1, 37.9) |
| West | 29.4 | 30.9 (28.1, 33.6) |

*Abbreviations: CI, Confidence Interval; DAST-10 Item, Drug Abuse Screening Test-10; GED, Graduate Equivalency Degree; IQR, Interquartile Range; USD, United States Dollar*

**Supplemental Figure 2. Recruitment flowchart for second wave of enriched sample used in replication of transport weights**

**
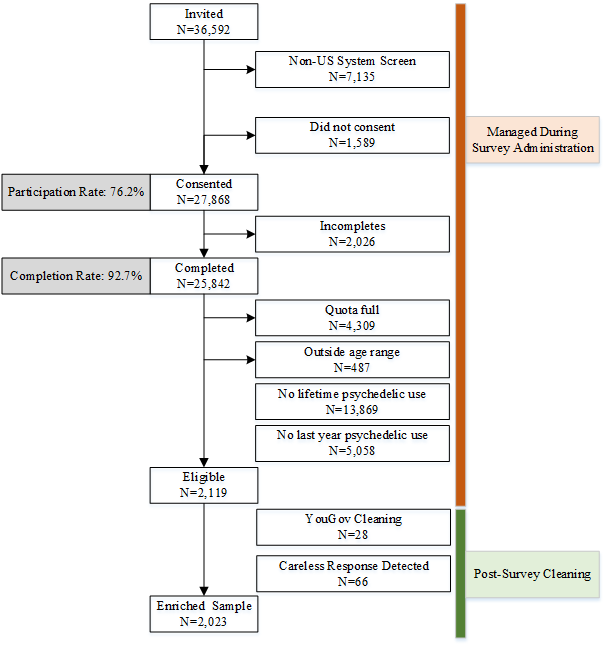
**

**Supplemental Figure 3. Changes in psychedelic use subpopulation characteristics after transport weighting, internal consistency in first wave.** Differences in anchor and fused subpopulations of psychedelic substance use in the last year showed little differences in demographic proportions, with acceptable but more notable changes in the health and substance use proportions. GED, Graduate Equivalency Degree


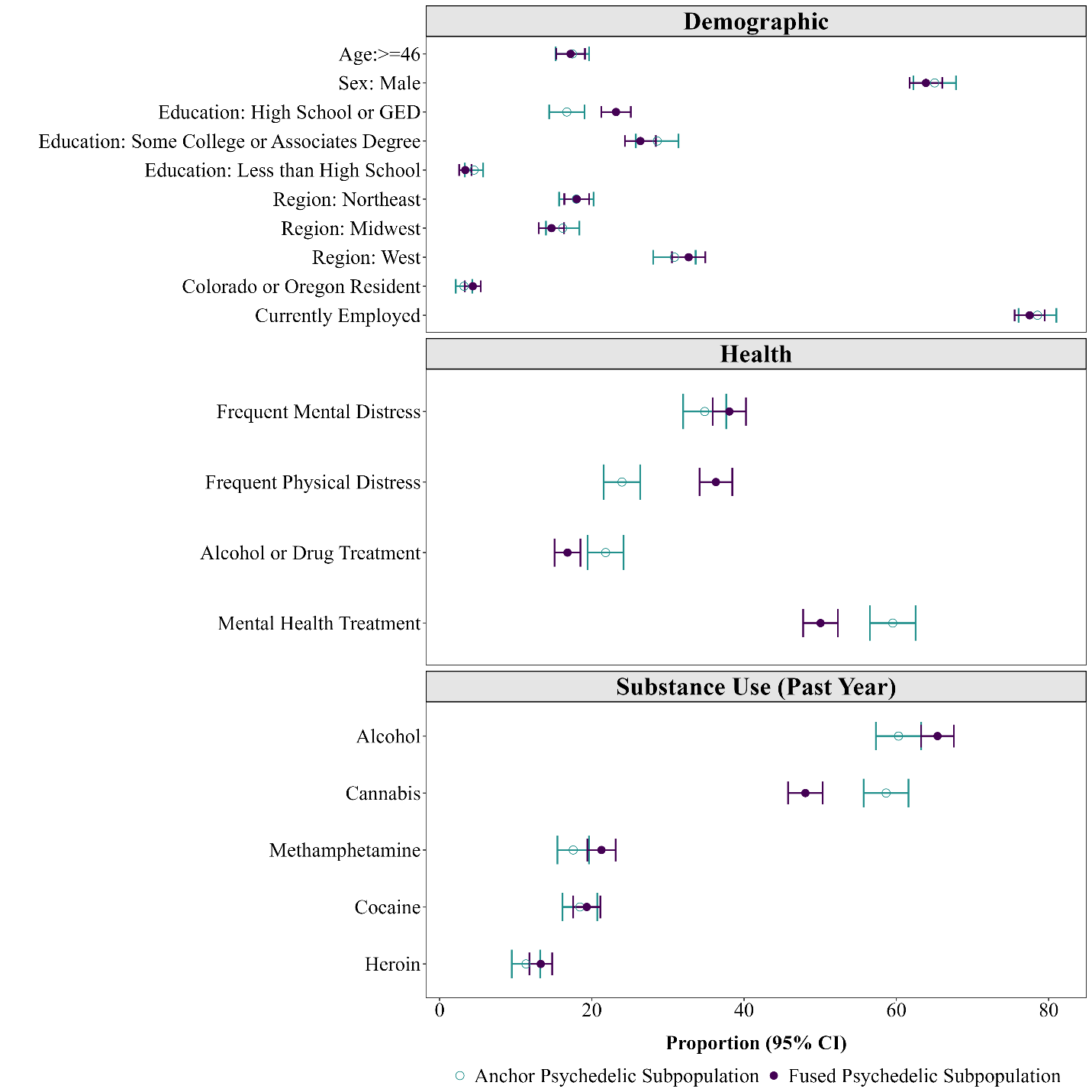


**Supplemental Figure 4. Changes in psychedelic use subpopulation characteristics after transport weighting, internal consistency in second wave.** Differences in anchor and fused subpopulations of psychedelic substance use in the last year showed similar internal consistency as the first wave after data fusion. There were small differences in demographic proportions, with less notable changes in the health and substance use proportions as the first wave.


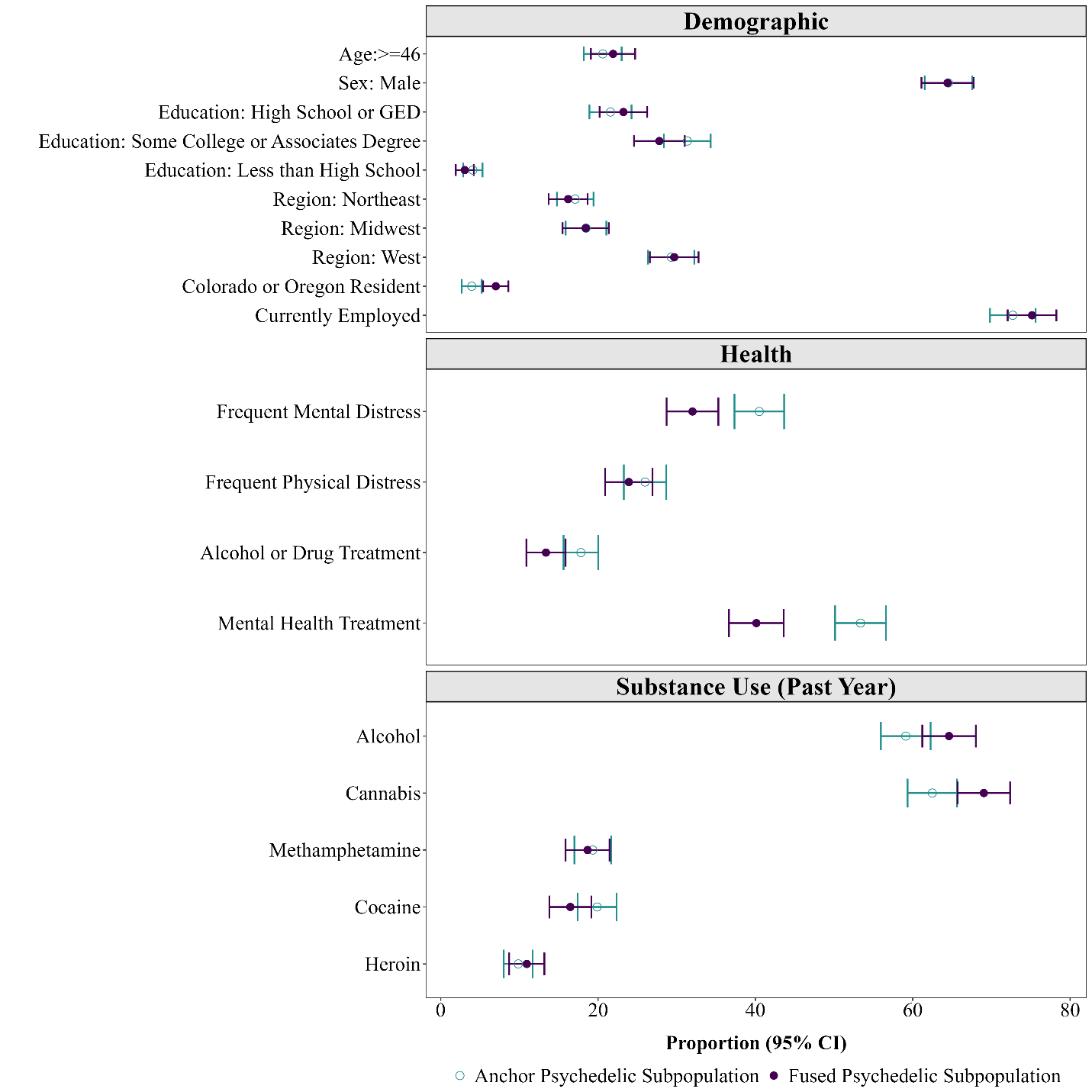


**Deviations to the Pre-Registered Statistical Analysis Plan on the Open Science Framework**

There were small deviations from the pre-registered analysis plan upon the empirical application. First, the transport variables were updated to use the most comparable estimates (for example replacing lifetime tobacco use with current cigarette use). This reduced any measurement error that might exist between the surveys (central assumption five). For the external evaluation variables, the National Health and Nutrition Examination Survey (NHANES) was replaced as the substance use gold standard since there were temporal differences in publicly available online data (latest was 2018). These were replaced by the National Survey of Drug Use and Health (NSDUH) 2023 data.

Our second hypothesis, that choice of the transport fusion anchor dataset will impact external validity was ultimately not able to be evaluated due to violations in the assumptions. When we attempted to identify the subpopulation of psychedelic use in NSDUH, there were substantial inconsistencies in the subpopulation definition compared to NSIHT. In the first wave of NSIHT when data fusion was attempted, 30.8% of NSIHT respondents endorsed a psychedelic other than those available in NSDUH. This grossly violated central assumption two and therefore we did not proceed with fusion. Ultimately, the alignment of the anchor dataset and enriched dataset proved very important in application.
